# Primary Dural Lymphoma: Clinical Cases and Literature Review

**DOI:** 10.1155/2020/2812487

**Published:** 2020-04-21

**Authors:** M. Dobran, R. Paracino, F. Mancini, D. Nasi

**Affiliations:** Department of Neurosurgery Azienda Ospedali Riuniti, Università Politecnica Delle Marche, Ancona, Italy

## Abstract

Primary dural lymphoma (PDL) is an extranodal non-Hodgkin lymphoma that accounts for less than 1% of all central nervous system lymphomas. Primary dural lymphoma grows from the dura mater, and it is often diagnosed as meningioma or acute subdural hematoma due to its radiological characteristics. Surgery is the gold standard of therapy; in many patients, PDL is relatively benign with good outcome. Authors report their experience in three patients affected by extranodal non-Hodgkin lymphoma (PDL) mimicking a meningioma.

## 1. Introduction

Primary dural lymphoma (PDL) is an extranodal non-Hodgkin lymphoma, a subtype of primary central nervous system lymphoma (PCNSL). This lymphoma develops primarily from brain parenchyma, eyes, meninges, or spinal cord in the absence of systemic disease. This lymphoma subtype accounts less than 1% of all central nervous system lymphomas and less than 0 1% of all non-Hodgkin's lymphomas [1, 2]. The central nervous system is involved either as a secondary spread of a systemic disease (5–10% of all cases) and as a primitive lesion in 1–2% [3, 4]. The literature reports only one series of PDL with 8 patients and another one with 15 cases [3–5]. PDL grows from the dura mater, and usually it is a low-grade marginal zone B-cell lymphoma (MZL). PDL is very rare, and it is often diagnosed as meningioma or acute subdural hematoma causes its radiological characteristics [6, 7]. The prognosis of primary malignant dural B-cell-type lymphoma is relatively benign and may be treatable by surgical resection with or without postoperative focal radiotherapy. We report our experience in three cases of extranodal non-Hodgkin lymphoma of dura mater (PDL) mimicking a meningiomas.

## 2. Case Report

**Figure 1:**
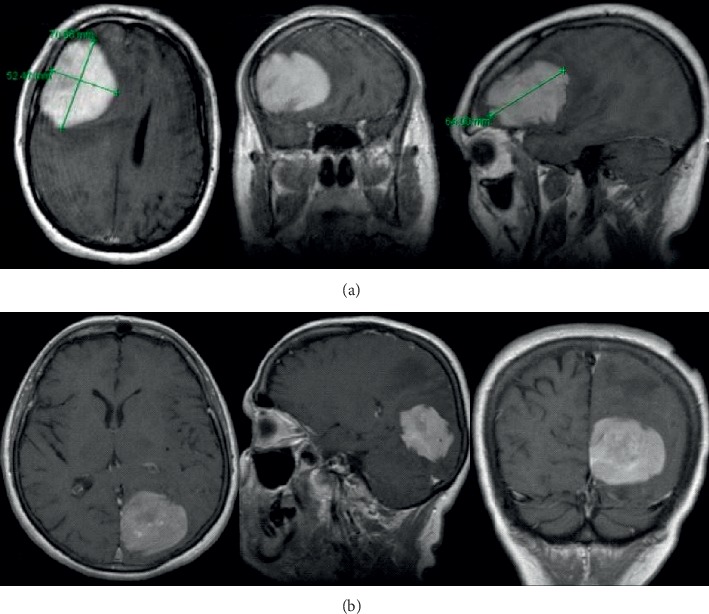
MRI study with contrast documented the presence of expansive lesions and isointense on T1-weighted, and hyperintense on T2-weighted images, characterized by an intense post-contrastographic enhancement, with dural tail, located in the first case in the right frontal region (a) and in the second case in the left occipito-parietal region (b).

**Figure 2:**
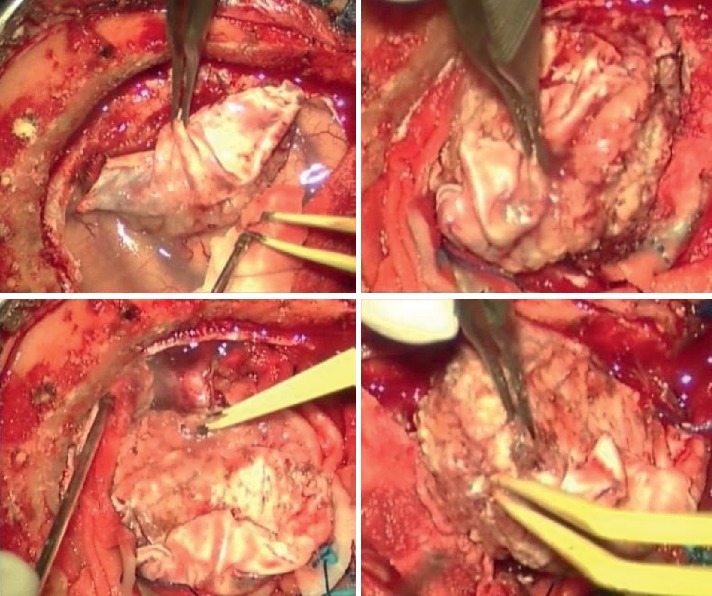
Intraoperative image of first case: the lesions, even though adherent to the dural structures, appeared with infiltrative characteristics, variable consistency (pseudofibrous shoots), intense vascularization, and bleeding.

**Figure 3:**
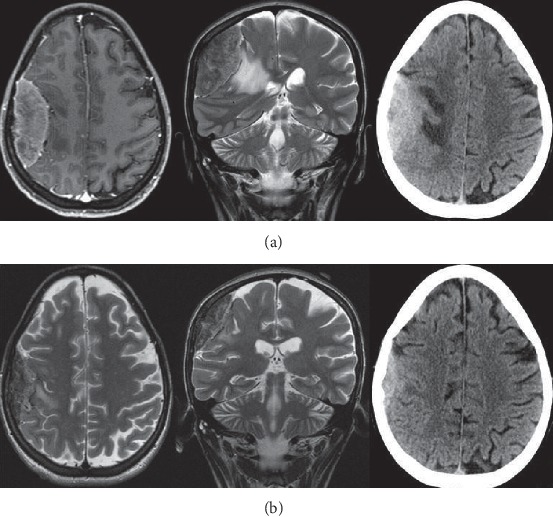
(a) MRI study with enhancement documented right fronto-parietal dural-based extra-axial mass causing moderate mass effect. The lesion was isointense on T1-weighted and hyperintense on T2-weighted images, with evidence of dural tail. (b) A mass reduction at CT-scan and MRI studies after 1 week of corticosteroid therapy.

**Figure 4:**
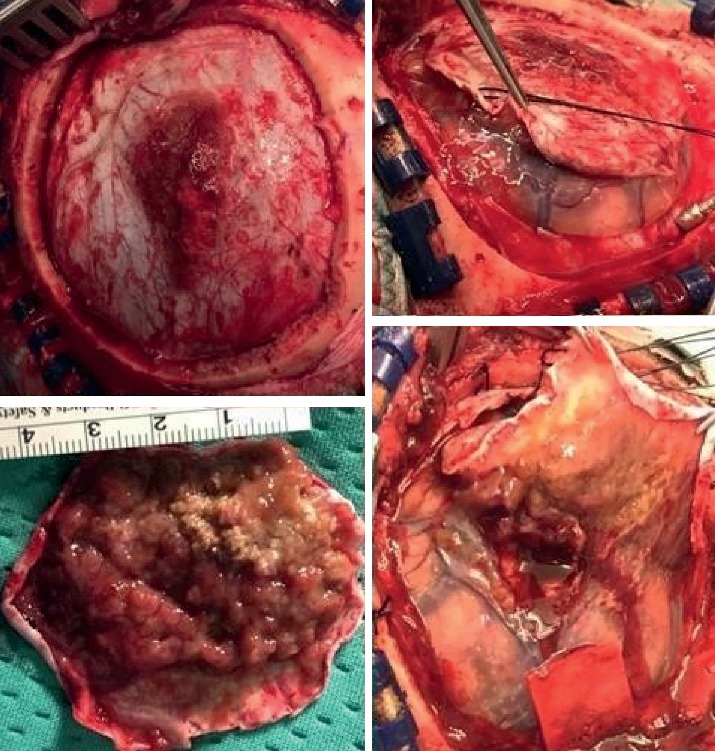
Right frontal craniotomy was performed with macroscopic excision of the tumor adherent to the dura with infiltrative characteristics a pseudofibrous consistency.

From 2012 to 2018, in the Clinic of Neurosurgery of Ancona three patients affected by primary dural lymphoma have been admitted. The first patient was a 49-year-old man with clinical history of personality and mood change and the second a 64-year-old woman with onset of right-handed and lateral hemianopsia. An MRI study with contrast documented the presence of expansive lesions with surrounding edema and isointense on T1-weighted and hyperintense on T2-weighted images, characterized by an intense post-contrastographic enhancement, with dural implant, located in the first case in the right frontal region, and in the second case, in the left occipito-parietal region (Figure 1). Due to the intracranial lesion position and imaging characteristics, a typical meningioma was suspected in both cases. Both patients underwent a craniotomy with complete macroscopic excision of the tumor. Intraoperatively, the lesions, even though adherent to the dural structures, appeared with infiltrative characteristics, variable consistency (pseudofibrous shoots), intense vascularization, and bleeding (Figure 2), but complete excision of tumor was performed in each case. Histology documented dense polymorphous lymphoid proliferation and large lymphocytes were found. Immunohistochemical study revealed specific antibody for B-cell lymphocytes in tumor cell membranes (CD20+, CD3−, CD30−, EMA−, CD79a+, MUM1+, IgMcit +, IgD−, CD10−, CD5−, Tdt−, Cd34−, BCL6, and BCL1 negative). The MIB-1 index was 15% in the first case and 20% in the second. These pathological features were compatible in each case with malignant lymphoma of diffuse large B-cell type. No extra cranial tumor was identified in further radiological exams in the two patients. In 2018, we observed a 26-year-old woman admitted at our department with one-month history of left arm weakness. Neurological examination was negative. A cerebral MRI study with enhancement documented a solitary right fronto-parietal dural-based extra-axial mass causing moderate mass effect. The lesion was isointense on T1-weighted and hyperintense on T2-weighted images, presenting homogenous enhancement, with evidence of dural tail. Imaging characteristics were consistent with right fronto-parietal convexity meningioma (Figure 3), but a mass reduction of the lesion at CT-scan after 1 week of corticosteroid therapy raised a doubt (Figure 3). Right frontal craniotomy was performed with macroscopic excision of the tumor adherent to the dura with infiltrative characteristics a pseudofibrous consistency (Figure 4). The postoperative cranial wound infection was treated with antibiotics therapy without bone flap plates removal and complete recovery. Histopathological analysis documented lymphocytic and plasmacytoid cells, consistent with a diagnosis of PDL. Immunostaining was CD20+, CD3−, CD30−, EMA−, CD79a+, MUM1+, IgMcit+, IgD−, CD10−, CD5−, Tdt−, Cd34−, BCL6, and BCL1 negative, Mib-1 5%. Postoperatory staging studies PET and total body CT were negative. Only in the first case, the patient underwent adjuvant radiotherapy treatment. In the second and third cases, the watch and see option was chosen with radiological controls. Both patients are currently alive without recurrence, while in the first case, the patient died in 2015 due to complications not related to cranial pathology.

## 3. Discussion

Primary dural lymphoma (PDL) is a very rare disease, and most commonly it occurs in the 4^th^–6^th^ decade of life predominantly in women. The incidence of this tumor varies from 0.6 to 3% of all brain neoplasms [6–9]. Iwamoto et al. reported an incidence about 2.4% on 355 patients with primary CNS lymphoma in their institution [4, 5]. Meningeal involvement of lymphoma, as a result of systemic disease, can occur extremely rare. For patients with PDL the most predominant histopathological finding is a low-grade marginal B cell lymphoma (MZL).

Primary dural lymphoma (PDL) should be considered in the differential diagnosis of meningiomas. In O'Neil series of 15 patients with PDL, a clinical and radiographic diagnosis of meningioma was made in 14 out of 15 patients prior histopathological diagnosis [3]. The PDLs share many features, including higher incidence in women and frequent occurrence of more than one extrassial lesion. When suspecting a PCNLS, enhanced MRI is the exam of choice, but neuroimaging findings are similar with meningiomas: both tumors present with extra-axial lesions that appear iso-hypointense on T1-weighted MR images and diffusely enhance with gadolinium. Moreover, dural tail sign, en plaque thickening of meninges, calvarial hyperostosis, and bone erosion have been common finding in both, but underlying vasogenic edema appears most common in PDL and depending on mass size and location [1, 3, 10]. Cerebral convexities are the most common site of involvement, but other sites are falx, tentorium, sellar, and suprasellar regions. Other differential diagnoses include dural metastasis, fibrous tumors, gliosarcomas, leiosarcomas, or neurosarcoidosis.

Primary dural lymphoma is a rare disease, and no standard treatment is described in literature. Surgical total excision remains the gold standard of treatment. Because complete resection of PDL may be technically difficult due to multiple tumors and infiltrative and en plaque presentation, many adjuvant drugs were indicated: systemic or intrathecal chemotherapy with R-CHOP, high dose of methotrexate, cytarabione, and vincristine [8–10]. As reported in the retrospective analysis by Quinn et al., given the rarity of diagnoses and paucity of available data, an optimal treatment strategy is still under debate. If complete resection is achieved, clinical and radiological follow-up, with no additional treatment, is appropriate; however, in most cases, adjuvant treatment with either radio or chemotherapy is necessary. Adjuvant radiotherapy is preferable because PDL is very radiosensitive, and it requires relatively low doses of radiation [9]. High-dose metrothexate and R-CHOP are the most effective drugs for parenchymal CNS lymphoma (PCNSL), but the effect is unclear in PDL [1, 2, 9]. In conclusion, there is no guideline in the treatment of PDL, so the decision should be tailored on each patient, based on the immunological and histopathological characteristics of the lesion. If leptomeninge are involved, chemotherapy or whole brain irradiation is required. Finally, when postoperative cranial wound infection develops the antibiotic therapy with or without tissue debridement grant recovery as in other surgical infections; the titanium plates of the bone flap may be maintained, but other complications may occur (subdural hematoma, subdural hygroma, intraparenchimal hematoma, hydrocephalus) [11, 12]. A primary dural lymphoma has a better prognosis than parenchymal primary CNS lymphoma or systemic lymphoma with CNS metastasis. PDL are biologically indolent, with survival rates at 5 years around 70%, with positive response to surgery and adjuvant radiotherapy [1, 4, 6, 9].

## 4. Conclusion

Primary dural lymphoma is a very rare disease, and it should be considered in the differential diagnosis of other tumors of dura mater and/or cranial bone. Our cases illustrate that intracranial lymphoma may rarely present as a wide dural-based mass mimicking a meningioma clinically, radiologically, and intraoperatively. So it must be considered in the differential diagnosis for all extra-axial enhancing lesions. Since PDL is very rare, there is no standard treatment for this tumor, so the decision should be tailored on each patient, based on the immunological and histopathological features of lesion. However, surgical excision and radiotherapy are the gold standard of therapy which grant good outcome.
